# PTN from Leydig cells activates SDC2 and modulates human spermatogonial stem cell proliferation and survival via GFRA1

**DOI:** 10.1186/s40659-024-00546-6

**Published:** 2024-09-16

**Authors:** Xueheng Zhao, Lvjun Liu, Zenghui Huang, Fang Zhu, Huan Zhang, Dai Zhou

**Affiliations:** 1 Hunan Provincial Key Laboratory of Regional Hereditary Birth Defect Prevention and Control, Changsha Hospital for Maternal & Child Health Care Affiliated to Hunan Normal University, Changsha, Hunan 410000 China; 2 Institute of Reproduction and Stem Cell Engineering, School of Basic Medicine Science, Central South University, Changsha, Hunan 410000 China; 3 Reproductive & Genetic Hospital of CITIC-Xiangya, Changsha, Hunan 410000 China

**Keywords:** Spermatogonial stem cells, Proliferation, Apoptosis, Self-renewal, Pleiotrophin, Syndecan-2

## Abstract

**Background:**

Spermatogonial stem cells (SSCs) are essential for the maintenance and initiation of male spermatogenesis. Despite the advances in understanding SSC biology in mouse models, the mechanisms underlying human SSC development remain elusive.

**Results:**

Here, we analyzed the signaling pathways involved in SSC regulation by testicular somatic cells using single-cell sequencing data (GEO datasets: GSE149512 and GSE112013) and identified that Leydig cells communicate with SSCs through pleiotrophin (PTN) and its receptor syndecan-2 (SDC2). Immunofluorescence, STRING prediction, and protein immunoprecipitation assays confirmed the interaction between PTN and SDC2 in spermatogonia, but their co-localization was observed only in approximately 50% of the cells. The knockdown of SDC2 in human SSC lines impaired cell proliferation, DNA synthesis, and the expression of PLZF, a key marker for SSC self-renewal. Transcriptome analysis revealed that SDC2 knockdown downregulated the expression of GFRA1, a crucial factor for SSC proliferation and self-renewal, and inhibited the HIF-1 signaling pathway. Exogenous PTN rescued the proliferation and GFRA1 expression in SDC2 knockdown SSC lines. In addition, we found downregulation of PTN and SDC2 as well as altered localization in non-obstructive azoospermia (NOA) patients, suggesting that downregulation of PTN and SDC2 may be associated with impaired spermatogenesis.

**Conclusions:**

Our results uncover a novel mechanism of human SSC regulation by the testicular microenvironment and suggest a potential therapeutic target for male infertility.

**Supplementary Information:**

The online version contains supplementary material available at 10.1186/s40659-024-00546-6.

## Background

Approximately 15% of couples in their reproductive years are impacted by infertility, with male factors contributing to 50% of the instances [1, 2]. Azoospermia, a severe form of male infertility, has a complex pathogenesis that is poorly understood and lacks effective treatments [3]. Hence, the management of individuals suffering from azoospermia, particularly non-obstructive azoospermia (NOA), poses a significant hurdle for medical professionals and scientists.

Promising methods for restoring male fertility in mice include the transplantation of spermatogonial stem cells (SSCs) and in vitro spermatogenesis [4]. SSC transplantation can regenerate in vivo spermatogenesis and overcome germ cell defects [5]. However, human and mouse SSCs differ in their regulatory mechanisms [6], which limits the research and translation of human SSCs. Therefore, it is crucial to investigate and clarify the elements that govern the destiny of human SSCs, as this is vital for human SSC transplantation and research on spermatogenesis in the laboratory.

SSCs are essential for maintaining a stable stem cell pool that supports continuous sperm production [7, 8]. The seminiferous tubule base houses these specific cells that undergo self-renewal and differentiation, which are controlled by intrinsic factors and testicular niches [9]. The destiny of SSC is influenced by its specific habitat and inherent characteristics. Various models have been used to study the self-renewal and differentiation of SSCs in mice, uncovering important pathways and molecules that play a role in determining their fate. Intrinsic factors such as PLZF [10], ID4 [11], and NANOS2 [12] enhance SSC self-renewal, while SALL4 [13] and RARγ [14] induce differentiation. Likewise, niche-derived growth factors such as GDNF [15], FGF [16], and EGF [17] facilitate self-renewal, whereas RA [18] and WNT [19]promote SSC differentiation. Notably, many reports suggested that melatonin also promotes the proliferation and self-renewal of SSCs [20, 21]. Moreover, recent studies suggest that SSCs are mainly located near the interstitial tissue and vasculature, possibly influenced by some molecules secreted by the vasculature [22].

While mouse models have provided insights into the control of rodent SSC self-renewal and differentiation [23], numerous discoveries cannot be extrapolated to humans because of variances in molecular and cellular aspects [24]. Few studies have reported on human SSC proliferation, self-renewal, and apoptosis. microRNA-1908-3p enhances SSC proliferation by degrading KLF2 [25]. FGF5 stimulates human SSC proliferation by activating AKT and ERK [26]. We have also previously shown that ASB9 [27], TCF3 [28], MAGEB2 [29], and SPOCD1 [30] are specifically expressed in human SSCs and regulate their self-renewal, proliferation, and apoptosis. Nevertheless, the processes regulating human SSC formation are still unknown.

Using single-cell data sets from GSE149512 [31] and GSE112013 [32], we examined the communication between niche cells and SSCs in this investigation. We detected high expression of Syndecans 2 (SDC2) in human SSCs and its ligand, Pleiotrophin (PTN), mainly in Leydig cells, and confirmed their interactions using immunofluorescence, STRING prediction, and protein immunoprecipitation assays. SDC2 knockdown markedly inhibited human SSC line proliferation and self-renewal marker expression. Exogenous PTN partially rescued the phenotypic defects caused by SDC2 deficiency. The results of RNA sequencing showed that the suppression of SDC2 led to a decrease in the expression of GFRA1, an important molecule for the self-renewal of SSCs and also had an impact on the HIF-1 signaling pathway. Moreover, we observed abnormal PTN and SDC2 expression in some NOA patients, suggesting that PTN and SDC2 abnormalities may impair spermatogenesis. Our data uncovered a mechanism of Leydig cell-derived PTN regulation of SSC line proliferation and self-renewal, which provides new insights into the niche-mediated fate regulation of SSCs.

## Materials and methods

### Ethical statement and sample collection

The ethics committee of CITIC-Xiangya approved this study (LL-SC-2021-025), and all participants provided signed informed consent. We collected testicular tissues from 15 azoospermia patients aged 25 to 46 who underwent testicular biopsy, with ∼ 25 mg of tissue from each patient. To eliminate blood cells, we thoroughly rinsed the samples using sterile PBS on a minimum of three occasions. Subsequently, we either preserved them in liquid nitrogen or treated them with 4% PFA or Bouin’s fixative solution.

### Analysis of cellular communication using data from single-cell RNA sequencing

Using the Read.10X function, we imported the expression matrix data into R and generated the Seurat object to analyze single-cell RNA sequence **(**scRNA-seq) datasets (GSE149512 and GSE112013) of normal adult testis. This was done with the assistance of Seurat 5, which can be found at https://github.com/satijalab/seurat/. Then, we filtered the gene expression data. In short, we kept cells that had gene expression values ranging from 500 to 4000 and fewer than 20% of genes related to mitochondria. We removed all mitochondrial and ribosomal genes based on their nomenclature. Using the R package DoubletFinder (https://github.com/chris-mcginnis-ucsf/DoubletFinder), we detected and eliminated duplicate entries. Afterwards, we applied the NormalizeData and FindVariableFeatures functions to each Seurat object. By utilizing the FindIntegrationAnchors and IntegrateData functions, we combined all Seurat objects. After utilizing the default (Uniform manifold approximation and projection) UMAP technique, we performed data clustering and subsequently determined cell types by evaluating the expression of cellular markers. After identifying and clustering the cells, we used the CellChat R package (http://www.cellchat.org/) to explore intercellular communication. In summary, we imported the metadata in the Seurat object into the CellChat program to create the CellChat object. Then, we used the CellChatDB.human database to identify and analyze receptor and ligand expression information in human testicular cells. We simulated the intercellular communication probabilities using the computeCommunProb function and integrated all potential signaling pathways and communication networks. After that, we used netVisual_circle to visualize cellular communication networks and netVisual_bubble to visualize all signal communications between the selected cells. In order to investigate the expression of SDC2 during the development of SSC, we collected data from spermatogonia, performed re-clustering using Seurat, and then imported the data into the Monocle3 R package (https://cole-trapnell-lab.github.io/monocle3/) to create developmental trajectories for spermatogonia. We created and modified all the dot, line, and violin plots using ggplot2 (https://github.com/tidyverse/ggplot2) in R.

### Culture of human SSC lines

The Human SSC line was created by introducing the Large T antigen into GPR125-positive undifferentiated spermatogonia of humans. The human SSC lines maintained numerous characteristics and indicators of primary SSCs, such as GFRA1, RET, and PLZF, while not exhibiting the presence of testicular endosomal cell markers like SOX9 [28, 33]. The immortalized human SSCs were cultivated at a temperature of 34 °C with a 5% concentration of CO2 in DMEM/F12 (Gibco, Carlsbad, CA, USA), which was enhanced with 10% FBS (Gibco). Cells were subcultured every 48 to 72 h using 0.5 g per liter of trypsin and 0.53 millimoles per liter of EDTA from Invitrogen. SSC lines were treated with PTN (50 ng/ml, Sinobiological, Beijing, China) to determine its effect on proliferation, self-renewal, and apoptosis.

### The process of extracting total RNA, performing reverse transcription PCR, and conducting quantitative PCR

Following the manufacturer’s instructions, we used RNA iso Plus reagent (Takara, Tokyo, Japan) to extract total RNA from isolated cells. The quality and concentration of the extracted RNA were assessed using Nanodrop from Thermo Fisher Scientific. Commercial kits (Roche, Basel, Switzerland) were utilized for the reverse transcription of cDNA. According to the instructions provided by the manufacturer, we conducted qPCR utilizing the ABI Prism 7700 system manufactured by Applied Biosystems. To determine the relative levels of mRNA, we employed the 2-△△(Ct) technique, with actin beta serving as an internal reference. After conducting a thorough analysis of every sample, we performed three repetitions and calculated the average of the outcomes. All primers were obtained from PrimerBank (https://pga.mgh.harvard.edu/primerbank/), and their sequences can be found in Table S1.

### Immunohistochemistry and immunofluorescence for tissue sections

The testes sections were deparaffinized with xylene and rehydrated with graded ethanol for immunohistochemistry. Next, we conducted heat-induced antigen retrieval by immersing the samples in a 0.01 mol/L sodium citrate buffer and heating them at 98°C for a duration of 18 minutes. After cooling and washing, we incubated the sections with 3% hydrogen peroxidase (Zsbio, Beijing, China) to block endogenous peroxidase activity. Following three rinses using phosphate buffer saline (PBS), the tissue sections were treated with 0.25% Triton X-100 (Sigma, St. Louis, MO, USA) for 15 minutes to make them permeable. Subsequently, nonspecific antigens were blocked by incubating the sections in 5% bovine serum albumin at room temperature for one hour. Afterwards, we incubated sections overnight at 4°C with the primary antibodies mentioned in Table S2. Following three rinses with PBS, the sections were then treated with horseradish peroxidase-conjugated goat anti-rabbit secondary antibody and incubated at room temperature for one hour. The color development was achieved using the 3,3’-diaminobenzidine chromogen kit (Dako, Glostrup, Denmark). The nucleus was stained with hematoxylin for a duration of 7 min at room temperature. In immunofluorescence, the primary antibody was incubated at 4 °C for 16 h, followed by chromogenic development using Alexa Fluor conjugated secondary antibody. Additionally, the cell nuclei were counterstained with 4’,6-diamidino-2-phenylindole (DAPI). We captured and analyzed microscopic images of testicular sections using a Zeiss microscope (Carl Zeiss, Jena, Germany).

### Western blotting and immunoprecipitation

The testicular tissue and cells were lysed with radio immunoprecipitation assay lysis buffer (RIPA, Thermo Fisher Scientific, Waltham, MA, USA) on ice for 15 min. After centrifugation at 12,000 g for 15 min, the supernatants were collected for total protein extraction and Western blot analysis. According to the instructions provided by the manufacturer, the bicinchoninic acid (BCA) Kit was utilized for determining the overall protein concentration. Each sample was analyzed using sodium dodecyl sulfate-polyacrylamide gel electrophoresis and Western blot analysis, following the previously described method [27], with twenty micrograms of total protein. For immunoprecipitation, we incubated the total protein with primary antibody or rabbit IgG overnight at 4 ℃, followed by Protein G magnetic beads for 2 h. After three washes with washing buffer, we collected the supernatant from the magnetically separated beads by heating at 95 °C for 5 min and used it for Western blotting. The antibodies are listed in Table S2. To visualize the protein bands, a chromogenic solution with enhanced chemiluminescence (Thermo Fisher Scientific) was employed, and the resulting chemiluminescent signals were captured and analyzed using Fusion FX (Vilber Lourmat). The analysis of all samples was conducted thrice, and the average results were calculated.

### siRNA transfection

Zorin (Shanghai, China) designed and synthesized all siRNAs, with their sequences listed in Table S3. The immortalized human SSCs were transfected with siRNAs (100 nmol/L) using Lipofectamine 3000 (Life Technologies) as per the instructions provided by the manufacturer. Cells were collected 48 h after transfection to extract protein and RNA for PCR and Western blot analysis.

### Cell viability assay

We utilized the Cell counting kit-8 (CCK8, Dojindo, Kumamoto, Japan) to assess the viability of SSCs based on the guidelines provided by the manufacturer. Cells were incubated for three hours with a culture medium supplemented with 100 mL/L CCK-8 reagents. The absorbance at 450 nm was measured using a microplate reader from Thermo Fisher Scientific.

### 5-ethynyl-2’-deoxyuridine (EdU) incorporation assay

An EdU labeling kit (RiboBio, Guangzhou, China) was used to detect DNA synthesis. Human SSCs were seeded in 96-well plates (5000 cells per well) in a culture medium supplemented with 50 µmol/L EdU. Following a 12-hour incubation period, the cells were rinsed with Dulbecco’s modified eagle medium (DMEM) and immobilized using 40 g per liter of PFA. Glycine (2 mg/ml) was used to neutralize the cells, followed by permeabilization with 5 mL/L Triton X-100 for 10 min at room temperature. EdU was detected using the Apollo staining reaction buffer, while the cell nuclei were stained with DAPI. Microscopic images of EdU-positive cells were captured and analyzed using the Zeiss fluorescence microscope. A minimum of 500 cells were evaluated in each sample.

### Cell apoptosis assay

After being transfected with SDC2-siRNA for a period of 48 h, the cells were treated with trypsin/Ethylene diamine tetraacetic acid (EDTA), followed by two washes using ice-cold PBS. Then, at least 106 cells were resuspended in Annexin V binding buffer (BD Biosciences, San Jose, CA, USA) and incubated with 5 µL APC-labeled Annexin V for 15 min at room temperature (RT). Cells were treated with 10 µL of PI and incubated for 10 min prior to the assay. Cell apoptosis was assessed using the BD Biosciences C6 flow cytometer.

### RNA-seq

Cell total RNA was isolated using the Trizol reagent kit (Invitrogen, Carlsbad, CA, USA). The quality of RNA was assessed by employing the Agilent 2100 Bioanalyzer (Agilent Technologies, Santa Clara, CA, USA). Oligo (dT) beads enriched eukaryotic mRNA, and a Magnetic Kit (Epicentre, Madison, WI, USA) removed rRNA. A fragmentation buffer was used to break down the enriched mRNA, which was then reverse-transcribed using random hexamers. Next, the cDNA was generated and purified using a purification kit from Qiagen. This was followed by end repair, introduction of poly (A), and ligation. The ligation products, which underwent PCR amplification, were separated using agarose electrophoresis and subsequently sequenced on the Illumina HiSeq2500 system. The reads from the sequencing machine were filtered by Fastp (0.18.0). The reads that were aligned to rRNA were eliminated by Bowtie2 (2.2.8). Transcripts were assembled, gene abundance was determined, and mapping to the reference genome was performed using the remaining clean reads. The mapped reads were assembled using a reference-based strategy by StringTie (version 1.3.1). DESeq2 software assessed differentially expressed genes (DEGs). In R, the ClusterProfiler package (https://github.com/YuLab-SMU/clusterProfiler) conducted enrichment analysis for DEGs using Gene Ontology and the Kyoto Encyclopedia of Genes and Genomes.

### Statistical analysis

The R programming language utilized the dplyr packages (https://dplyr.tidyverse.org) for conducting data analyses. Each experiment was conducted at least three times. The data is presented as the average plus standard deviation. The t-test was used to assess the variances among the groups. A significance level of less than 0.05 denoted statistical significance.

## Results

### Cell communications analysis of human testicular cells

**Fig. 1:**
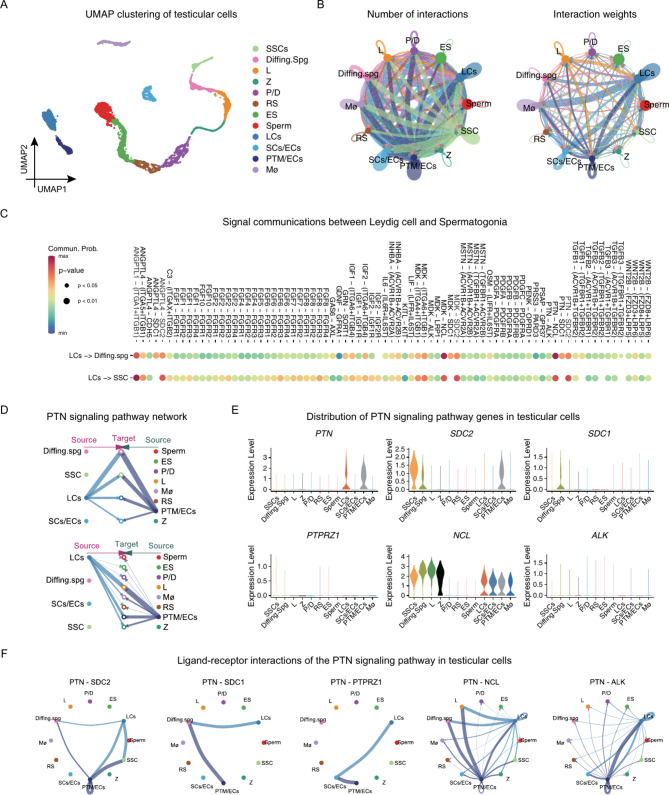
Single-cell sequencing reveals cellular communication among testicular cells. (**A**) UMAP cluster analysis of human testicular scRNA-seq data showing the distribution of different testicular cell types in two dimensions. (**B**) Total number and weight of cellular communication between testicular cells, with the direction and thickness of the arrows reflecting the direction and strength of intercellular signaling, respectively. (**C**) Dot plot showing the cellular signals emitted by Leydig cells towards SSCs and differentiating spermatogonia, with redder colors indicating a higher probability of that signaling pattern, and the dot size indicating the p-value. (**D**) Ligand and receptor circle plot showing the PTN signaling pathway between testicular cells, the thickness of the line represents the strength of the signaling. (**E**) Violin diagram showing the distribution of conventional PTN signaling pathway molecules in the testis. (**F**) Ligand and receptor interactions of the PTN signaling pathway in testicular cells. SSCs: spermatogonial stem cells, Diffing. Spg: differentiating spermatogonia, L: leptotene spermatocytes, Z: zygotene spermatocytes, P: pachytene spermatocytes, D: diplotene spermatocytes, RS: round spermatids, ES: elongated spermatids, LCs: Leydig cells, SCs: Sertoli cells, ECs: endothelial cells, PTM: peritubular myoid cells, Mø: macrophages

In order to clarify how the testicular microenvironment controls the fate determination of SSCs, we conducted an analysis of intercellular communication using scRNA-seq datasets obtained from normal adult testes in GSE112013 and GSE149512. After filtering and integrating the data, we retained 4937 cells and 22,075 genes. We classified all testicular cells into 12 clusters using the UMAP method and identified them according to the expression patterns of a series of common marker molecules (Fig. 1A). We used ID4, PIWIL4, and NANOS3 to identify SSCs; KIT and STRA8 to identify differentiating spermatogonia (Diffing. Spg); SYCP1, SYCP3, and SPO11 are used to distinguish the various phases of spermatocytes, which include leptotene (L), zygotene (Z), pachytene (P), and diplotene (D); SUN5, ZPBP, and TXNDC2 are employed to recognize the different stages of spermatids, such as round spermatids (RS) and elongated spermatids (ES); TNP2 and PRM2 are utilized to identify sperm; IGF1 and DLK1 are employed for the identification of Leydig cells (LCs); WT1 and SOX9 are used to distinguish Sertoli cells (SCs); VWF is utilized to identify endothelial cells (ECs); MYH11 is employed for the identification of peritubular myoid cells (PTM); CD68 is used to identify macrophages (Mø). To further examine the communications among cells in the testicles, we utilized the CellChat program in R. By utilizing the CellChatDB.human database, and we computed the potential connections between ligands and receptors in every cell. The results showed that LCs, Sertoli cells (SCs) / endothelial cells (ECs), and PTM/ECs had more interactions with other cells, but LCs had the highest interaction weights (Fig. 1B and Fig. S1). Therefore, we explored the signals that LCs might send to germ cells, especially SSCs and diffing.spg. We listed all the major signal communications between LCs and SSCs and diffing. Spg to find signals associated with SSC proliferation and self-renewal. The results suggested that PTN signaling pathway had higher communication probabilities and differed between SSCs and Diffing. Spg (Fig. 1C). In the testis, PTN signaling was calculated to originate mainly from LC and PTM/ECs and to be delivered to SSC and Diffing.spg (Fig. 1D). PTN has been shown to bind to five common receptors: PTPRZ1, SDC1, SDC2, ALK, and NCL. We detected high expression of three of these receptors in the testes, with SDC2 predominantly expressed in SSCs, SDC1 mainly in differentiating spermatogonia, and NCL more widely distributed (Fig. 1E). Ligand-receptor interaction calculations revealed that PTN signals to SSCs mainly through SDC2 binding, whereas SDC1 mediates PTN signaling in differentiating spermatogonia. Furthermore, NCL also participates in PTN reception in differentiating spermatogonia and L. (Fig. 1F). These results implied that PTN participates in SSC development through SDC2.

### Co-localization and interaction of SDC2 and PTN in human testes

**Fig. 2:**
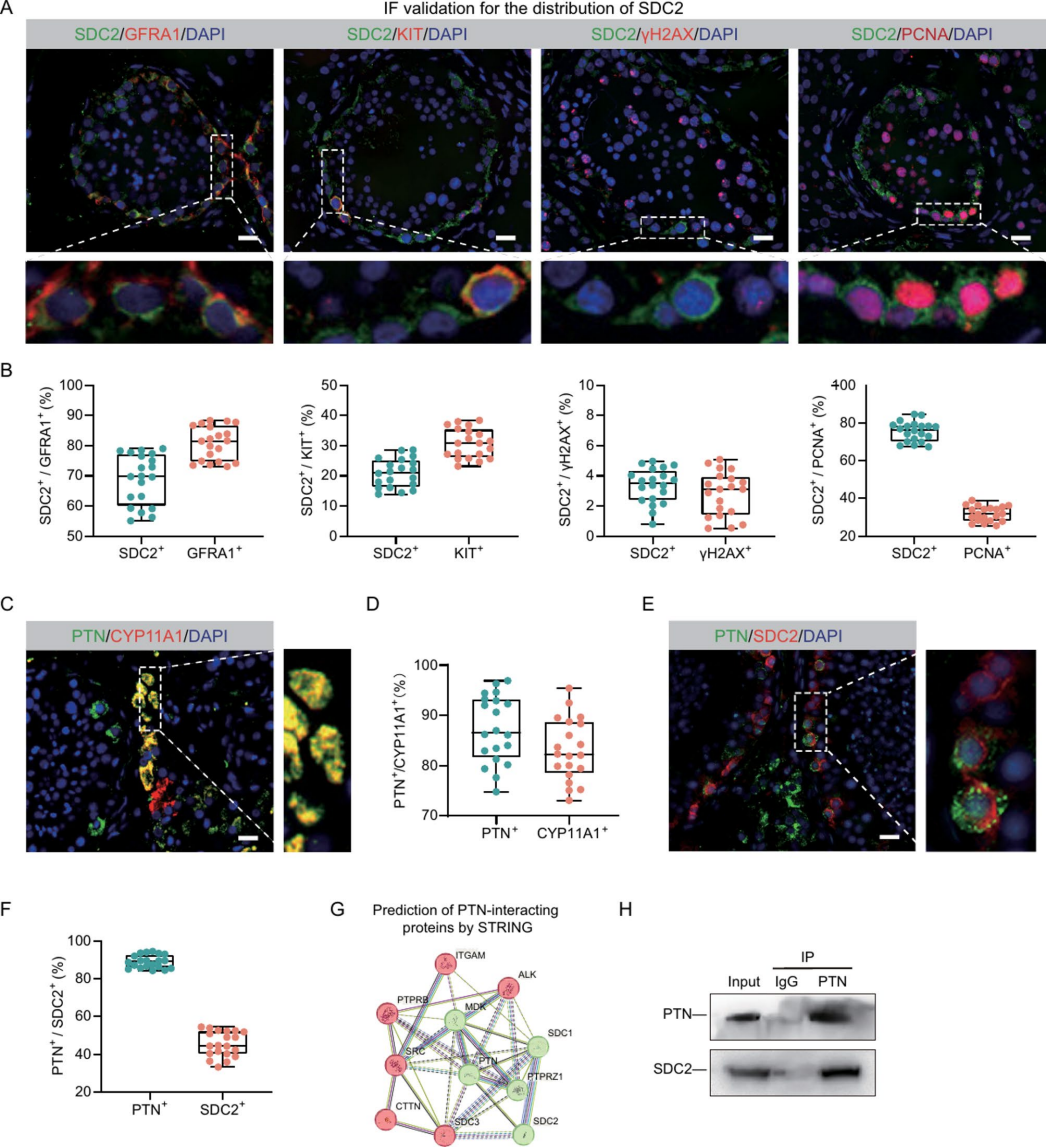
Expression patterns of PTN and SDC2 in normal human testicular tissues. (**A**) Double immunofluorescence localization of SDC2 with GFRA1, KIT, γH2AX and PCNA. Most SDC2-positive cells co-expressed GFRA1 and PCNA, and SDC2 rarely co-localized with KIT and occasionally co-localized with γH2AX. (**B**) Box plots showing the co-expression of SDC2 with multiple marker molecules in A. (**C**) Double immunofluorescence localization of PTN with CYP11A1 in testicular tissues. (**D**) Box plots showing the proportion of double-positive cells in C. (**E**) Double immunofluorescence localization of PTN with SDC2 in testicular tissues. (**F**) Box plots showing the proportion of double-positive cells in E. (**G**) The STRING database predicts reciprocal proteins for PTN, where SDC2 may have interactions with PTN. (**H**) Protein immunoprecipitation to detect the interaction of PTN and SDC2. The white dotted line in the fluorescent image indicates the edge of the seminiferous tubule. Scale bar, 50 μm

To investigate the potential roles of PTN and SDC2 in SSC fate determination, we analyzed the distribution pattern of SDC2 in spermatogonia developmental trajectories. All spermatogonia, including SSC and Diffing.spg were categorized into eight groups, spg_1 to spg_8, where spg_1 to spg_3 were considered SSCs because of their high expression of ID4, and spg_4 to spg_8 were considered Diffing. Spg because they lacked ID4 expression but highly expressed DMRT1 (Fig. S2A). Following this, we conducted developmental trajectory analysis with monocle3 and discovered that SDC2 exhibited predominant expression in SSCs during the initial stages of development. Furthermore, its expression gradually declined as spermatogonial differentiation occurred, indicating that SDC2 potentially plays a crucial role in the proliferation or maintains of SSCs (Fig. S2B and C). To confirm the findings of the bioinformatics analysis, we conducted a dual-color immunofluorescence (IF) assay utilizing various germ cell markers, such as GFRA1 (SSC marker), KIT (a marker indicating differentiated spermatogonia), γ-H2AX (a marker for late spermatocytes), and PCNA (a marker for actively dividing cells) (Fig. 2D). According to the findings, SSCs predominantly expressed SDC2, with approximately 68.93% ± 8.25% of SDC2-positive cells also expressing GFRA1. Additionally, around 28.88% ± 4.68% of SDC2-positive cells exhibited colocalization with KIT, while approximately 3% of spermatocytes displayed SDC2 expression. Moreover, it was observed that a majority of cells positive for SDC2 (approximately 75.51% ± 5.21%) exhibited PCNA expression, indicating its potential role in the self-renewal and proliferation of SSCs (Fig. 2A and B). Similarly, we found that PTN was predominantly distributed in the testicular mesenchyme and colocalized with CYP11A1, a marker of mature Leydig cells, and that about 83.14% of Leydig cells expressed PTN (Fig. 2C and D). We examined the localization of PTN and SDC2 using immunofluorescence, consistent with the bioinformatics results, PTN and SDC2 co-localized on SSCs, but only about 50% of SDC2-positive cells were bound with PTN (Fig. 2E and F). In addition, predictions from the STRING database and immunoprecipitation results of testicular proteins indicated an interaction between PTN and SDC2 (Fig. 2G and H). These results showed that PTN was mainly expressed in Leydig cells, while SDC2 was enriched in proliferating SSCs. PTN and SDC2 co-localized and interacted predominantly in SSCs, suggesting a possible binding between them that could modulate SSC self-renewal and proliferation.

### The influences of SDC2 knockdown on the proliferation and apoptosis of human SSC lines

**Fig. 3:**
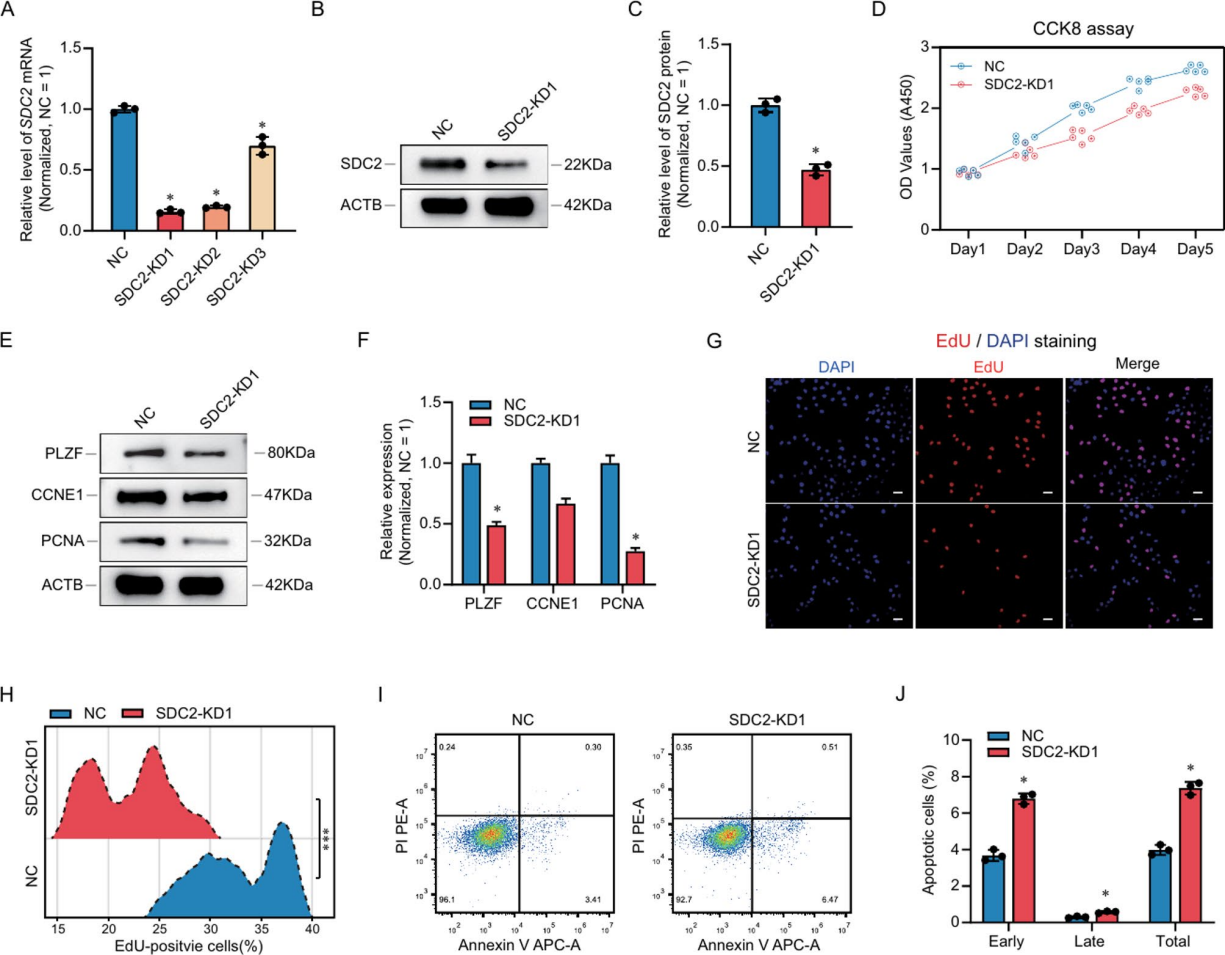
SDC2 knockdown impairs proliferation and self-renewal of human SSC lines in vitro. (**A**) qPCR analysis of SDC2 mRNA levels after small interfering RNA (siRNA) transfection. (**B**) Western blot analysis of SDC2 protein levels after knockdown. (**C**) Bar graphs showing the relative levels of SDC2 protein in the NC and knockdown groups, normalized to GAPDH. (**D**) CCK8 assay measuring cell proliferation, showing that SDC2 knockdown significantly inhibited SSC proliferation. (**E**) Western blot analysis of SSC self-renewal and proliferation-related proteins, including PLZF, CCNE1, and PCNA, showing that SDC2 knockdown significantly reduced the expression of these proteins. (**F**) Bar graphs showing the relative protein levels of PLZF, CCNE1, and PCNA in the NC and knockdown groups, normalized to GAPDH. (**G**) EdU assays assessing cellular DNA synthesis, showing that SDC2 knockdown significantly decreased the number of EdU-positive cells. (**H**) Ridgeline plots showing the distribution of EdU-positive cells in the NC and knockdown groups. (**I**) Flow cytometry combined with Annexin V to detect cell apoptosis, showing that SDC2 downregulation significantly increased cell apoptosis. (**J**) Bar graphs showing the apoptotic percentage of SSCs in the NC and knockdown groups. * indicates *P* < 0.05

To examine the functions of SDC2 in the proliferation of SSCs, we employed a cell line that possesses comparable traits to primary human SSCs. Due to the significant expression of SDC2 in testes, we employed small interfering RNA (siRNA) to suppress SDC2 and investigate its role in SSCs. We designed and synthesized three siRNAs and transfected them into the SSC line. Afterwards, we measured the SDC2 level using qPCR (Fig. 3A) and Western blot (Fig. 3B and C) and found that the expression of SDC2 was reduced after transfection with siRNAs, with the SDC2-KD1 group having the best knockdown efficiency. Furthermore, we assessed SSC proliferation after SDC2 knockdown using CCK8 and found that down-regulation of SDC2 significantly inhibited cell proliferation 3 to 5 days after transfection (Fig. 3D). Similarly, we noticed notable reductions in various proteins linked to SSC self-renewal and proliferation through Western blot analysis, such as PLZF, CCNE1, and PCNA (Fig. 3E). We also examined the cell DNA synthesis using an EdU assay 48 h after transfection and found that DNA synthesis of the SSC line was also significantly inhibited (Fig. 3F and G). Moreover, after SDC2 suppression, we found that apoptosis of the SSC line increased significantly, especially the early apoptosis (Fig. 3I and J). These data showed that inhibition of SDC2 affected SSC line proliferation and apoptosis, implying that SDC2 is necessary to stimulate SSCs.

### RNA sequencing and screening of downstream genes

**Fig. 4:**
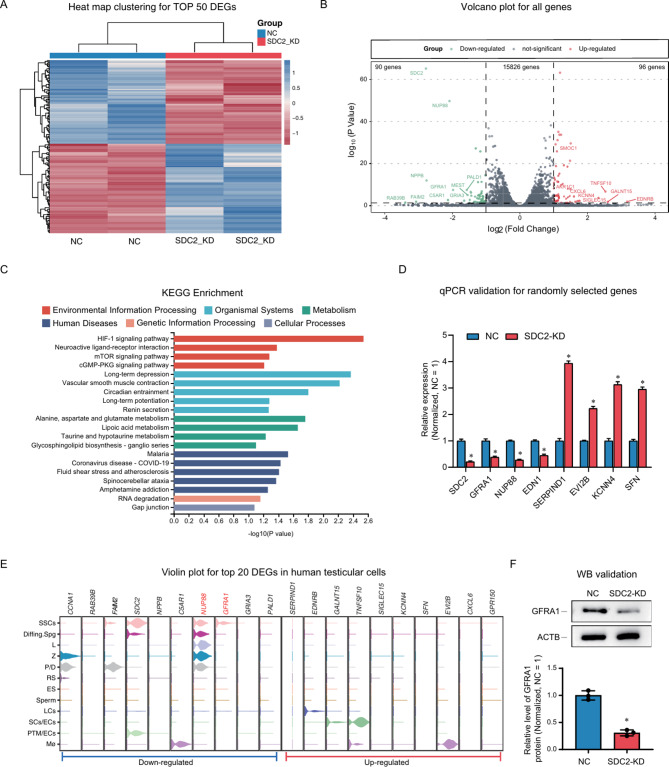
RNA sequencing identifies downstream genes of SDC2. (**A**) Heatmap of the top 50 DEGs showing the concordance of gene expression after SDC2 knockdown. (**B**) Volcano plot displaying the expression of all genes after SDC2 knockdown. 90 genes were significantly down-regulated and 96 genes were significantly up-regulated by SDC2 knockdown. Red color indicates up-regulated genes and green color indicates down-regulated genes. (**C**) KEGG enrichment analysis of all significantly down-regulated genes. (**D**) qPCR validation of randomly selected DEGs confirming the reliability of RNA sequencing. (**E**) Violin plot showing the distribution of the top 20 DEGs in human testis. GFRA1 and NUP88 are highly expressed in SSCs. (**F**) Western blot analysis verifying the differential expression of GFRA1 in the NC and knockdown groups

To further investigate SDC2-mediated signaling and downstream genes, we examined changes in the mRNA profile in the SSC line after SDC2 knockdown. We detected 16,012 genes using bulk mRNA sequencing. Among them, 90 genes were significantly down-regulated, and 96 genes were significantly up-regulated (fold changes > 2, *P* value < 0.05, Table S4). Using a heat map, we grouped the top 50 genes that showed DEGs and found that the levels of these genes were consistent among the different groups, suggesting a strong agreement among the samples (Fig. 4A). We used a volcano plot to show the expression distribution of all genes, and we colored all genes according to their variation (up-regulated: red, down-regulated: green, not significant: gray). We also labeled significant DEGs in the volcano plot (Fig. 4B). Then, we performed an enrichment analysis of all DEGs and down-regulated DEGs using Gene ontology (GO, Fig. S3) and Kyoto encyclopedia of genes and genomes (KEGG) databases (Fig. 4C) and found that SDC2 inhibition downregulated signaling pathways such as HIF-1 and mTOR. Additionally, a random selection of DEGs such as *SDC2*, *GFRA1*, *NUP88*, *EDN1*, *SERPIND1*, and *EVI2B* were chosen, and their levels were confirmed through qPCR. The results were consistent with RNA sequencing, indicating the reliability of RNA sequencing (Fig. 4D). Meanwhile, we examined the distribution of the top 10 down-regulated genes in the testis using scRNA-seq data and found that GFRA1 and NUP88 were abundantly expressed in human SSCs and were more likely to be potential downstream targets of SDC2 (Fig. 4E). We further confirmed the downregulation of GFRA1 using Western blot (Fig. 4F), suggesting that GFRA1 is a target of SDC2. Considering that the roles of GFRA1 on SSC are well known [15, 34, 35], we did not validate it. These data suggested that SDC2 down-regulation suppressed GFRA1 expression in SSC and inhibited the HIF-1 and mTOR pathways.

### PTN rescued the proliferation, apoptosis and GFRA1 expression of SDC2-deficient SSC lines

**Fig. 5:**
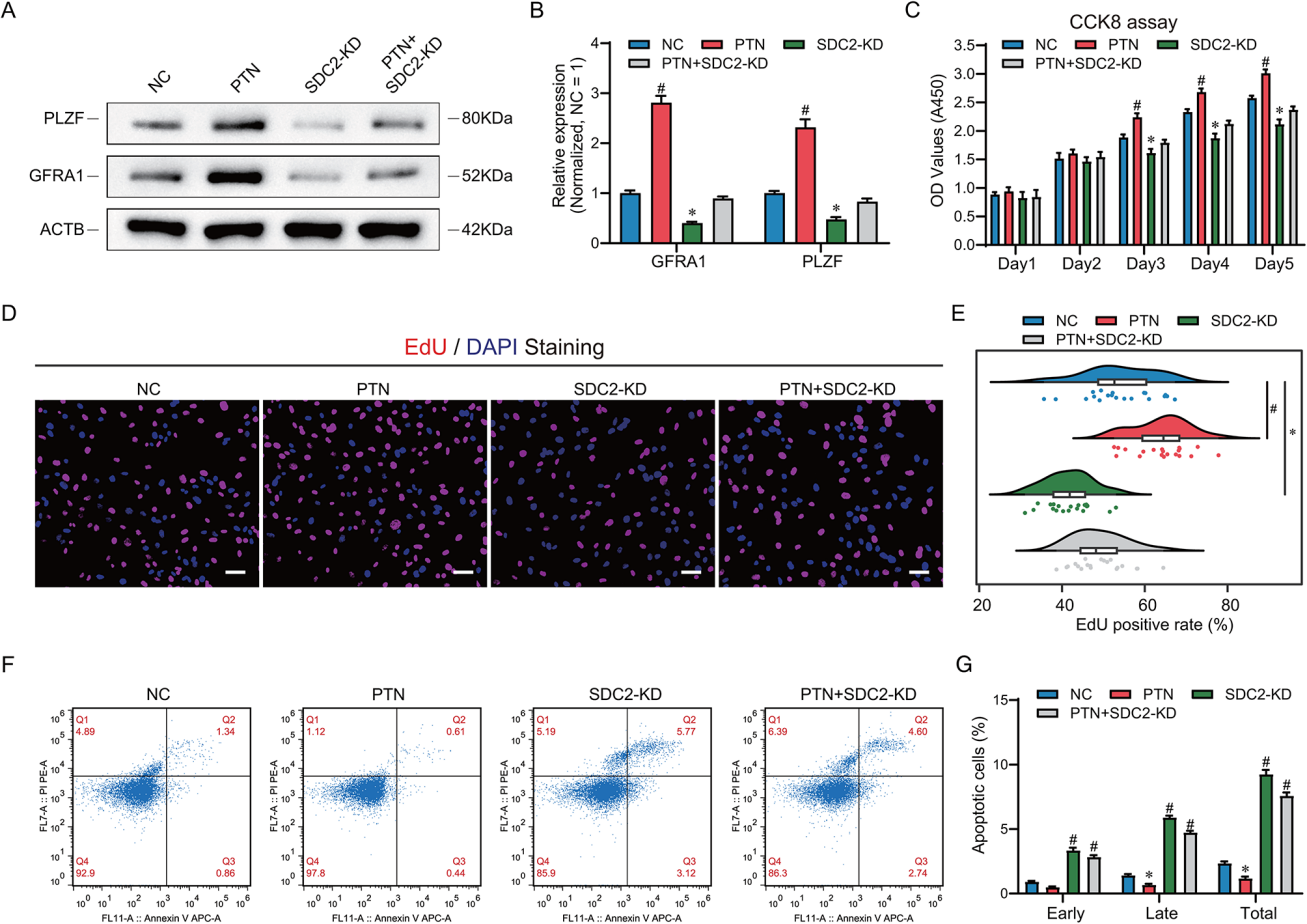
Exogenous PTN rescues the phenotypic defects induced by SDC2 deficiency. (**A**) Western blot analysis of PLZF and GFRA1 expression levels in the NC, PTN, SDC2-KD and PTN + SDC2-KD groups. The results showed that PLZF and GFRA1 expression was significantly increased in the PTN group compared with the NC group, while PLZF and GFRA1 expression was significantly decreased in the SDC2-KD group, and PLZF and GFRA1 expression in the PTN + SDC2-KD group was comparable to that of the NC group, suggesting that PTN could restore the expression of PLZF and GFRA1. (**B**) Bar graphs showing the relative levels of PLZF and GFRA1 in (A), normalized to the NC group. (**C**) CCK8 assay measuring cell proliferation in the four groups, indicating that exogenous PTN alleviated the proliferation defect caused by SDC2 knockdown. (**D**) EdU assays assessing cellular DNA synthesis in the four groups, showing that PTN supplementation rescued the DNA synthesis impairment induced by SDC2 deficiency. (**E**) Violin plots showing the EdU positivity rates of the four groups in (D). (**F**) Flow cytometry combined with Annexin V to detect cell apoptosis in the four groups, showing that PTN addition reduced the apoptosis induced by SDC2 deficiency. (**G**) Bar graphs showing the apoptotic percentage of SSCs in the four groups. * indicates significant down-regulation compared to the NC group, and # indicates significant up-regulation compared to the NC group, *P* < 0.05. Scale bar in (**D**) is 50 μm

Previous studies suggested that PTN is a multifunctional factor that promotes stem cell maintenance and proliferation [36, 37]. Considering that not all SSC bind PTN in the physiological state (Fig. 2E), we sought to test whether exogenous PTN promotes SSC proliferation and self-renewal in vitro and whether PTN can rescue the phenotypic defects downregulated by SDC2. The Western blot findings indicated that the introduction of PTN enhanced the levels of PLZF and GFRA1, which are crucial markers for SSCs, and restored their expression in SSC lines lacking SDC2 (Fig. 5A and B). CCK8 results showed that PTN stimulated SSC proliferation and rescued the proliferation impairment caused by SDC2 knockdown (Fig. 5C). EdU assay also demonstrated that PTN rescued DNA synthesis inhibited by SDC2 knockdown (Fig. 5D and E), consistent with CCK8 results. In addition, PTN significantly reduced apoptosis with or without SDC2 knockdown (Fig. 5F and G). These data indicate that PTN stimulates the growth of human SSC lines in vitro and rescues the phenotypic defects induced by SDC2 knockdown, as well as upregulates the expression of GFRA1, a downstream protein of SDC2. These findings indicated that PTN modulates SSC proliferation and apoptosis via SDC2.

### Aberrant expression of PTN and SDC2 may be associated with NOA

**Fig. 6:**
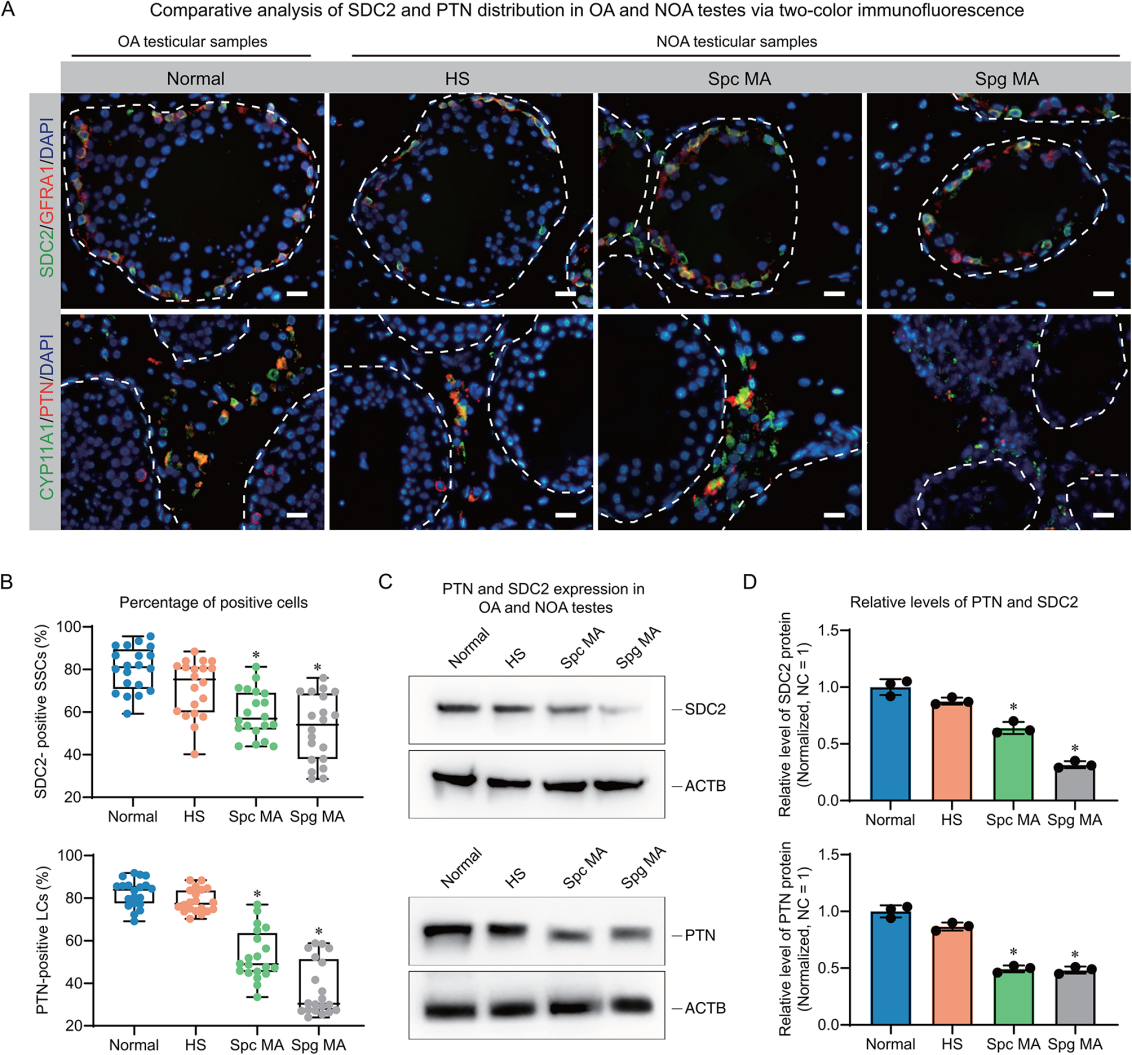
Expression of PTN and SDC2 in testicular tissues of patients with OA and NOA. (**A**) Double immunofluorescence localization of PTN and SDC2 in testicular tissues with varying spermatogenic potential. (**B**) Box plots showing the proportion of PTN- and SDC2-positive cells in different testicular tissues. (**C**) Western blot analysis of PTN and SDC2 protein levels in different testicular tissues. (**D**) Bar graphs comparing the relative expression of PTN and SDC2 in different testicular tissues. Normal denotes testicular tissues with normal spermatogenesis, HS denotes testicular tissues with hypospermatogenesis, Spc MA denotes testicular tissues with spermatocyte maturation arrest, and Spg MA denotes testicular tissues with spermatogonia maturation arrest. The scale bar in A is 50 μm, * indicates significant difference from the normal group, *P* < 0.05

We further explored the distribution of PTN and SDC2 in patients with NOA and found differences in the expression of PTN and SDC2 in some samples. The samples were classified based on the pathological features of the testes, which encompassed obstructive azoospermia (OA), hyperspermatogenesis (HS), spermatocyte maturation arrest (Spc MA), and spermatogonia maturation arrest (Spg MA). In certain individuals diagnosed with Spg MA and Spc MA, the SDC2-positive rate of SSCs (GFRA1-positive) showed a notable decline, as indicated by the results obtained from two-color immunofluorescence (IF) analysis. We also found reduced positive rates of PTN in Leydig cells in these samples. Interestingly, the number of PTN-bound SSCs also seems to be decreased, but we are still looking for statistical evidence (Fig. 6A and B). Western blot experiments yielded similar findings, with significant decreases in total levels of PTN and SDC2 in some of the NOA patients (Fig. 6C and D). These findings suggested that aberrant expression of PTN and SDC2 may be associated with the occurrence of NOA.

## Discussion

SSCs serve as the source of spermatogenesis and provide continuous support for the production of sperm throughout a male’s lifetime [8]. SSCs in mice possess the capacity to revive spermatogenesis in the receiving testis [5], offering a fresh approach to addressing male infertility, particularly NOA. Nevertheless, the lack of understanding regarding the regulatory mechanisms of human SSCs poses a hindrance to the implementation of stem cell therapy for male infertility. Intrinsic molecules and the testis microenvironment drive the fate decision of SSCs [23]. The testicular microenvironment is crucial for the preservation and specialization of SSCs. In general, the self-renewal and differentiation of SSCs are regulated by Sertoli cells, Leydig cells, and peritubular myoid cells through the secretion of growth factors and the provision of structural support, as commonly believed [38]. However, the molecular mechanisms by which they regulate SSCs remain poorly understood.

By analyzing cellular communication at the single-cell level in normal testicular cells, we found that PTN in the testicular niche might regulate SSCs through SDC2.PTN is a protein that is released and has an affinity for heparin, and it can attach to various receptors, including PTPRZ [39], Syndecans [40], and Nucleolin [41]. PTPRZ was the most extensively researched receptor for PTN. In glioblastoma, infiltrating macrophages secrete PTN. This activated Fyn and AKT signaling in glioblastoma stem cells via its receptor PTPRZ1, thereby promoting tumor growth as confirmed by studies [39, 42]. Additionally, PTN stimulated the proliferation of neural stem and progenitor cells by activating their AKT signaling pathway, a process mediated through PTPRZ1 [37]. Syndecans constitute a family of proteoglycans, encompassing four members: SDC1, SDC2, SDC3, and SDC4 [43]. It has been documented that PTN engages with Syndecans via the Glycosaminoglycan (GAG) domain [43]. Notably, PTN’s interaction with SDC3 triggers the activation of ERK1/2, thereby facilitating the metastasis of prostate cancer cells [44]. However, there is a paucity of research elucidating the downstream signaling pathway collaboratively modulated by PTN and SDC2. In several tumors, including gastric and colon cancers, SDC2 was overexpressed [45, 46]. This upregulation catalyzed the activation of signaling pathways such as AKT and ERK, fostering tumor growth and correlating with a less favorable prognosis [46–48]. Moreover, SDC2 is prevalent in hematopoietic stem cells, where it exerts influence over their self-renewal and quiescence via p57 [49]. In our scRNA-seq analysis, we identified another receptor for PTN, NCL, in the testis. However, we did not observe differential expression of NCL in SSCs. The role of PTN in regulating the fate decision of SSCs via NCL remains unclear. We also detected MDK expression in Leydig cells, differentiating spermatogonia and leptotene spermatocytes, but whether it plays a role in SSCs remains to be further tested. Using two-color immunofluorescence, we observed that PTN bound to only about 50% of SDC2-positive cells. The reason for the lack of binding in the remaining cells is unclear. It could be due to the sensitivity of the experiment or to the absence of PTN-SDC2 interaction. Further experiments are needed to confirm this.

Although there are no studies on PTN testicular conditional knockout mice, chimeric mice generated by introducing dominant-negative PTN mutants into embryonic stem cells using homologous recombination are sterile [50], suggesting that PTN has an important role in testicular spermatogenesis, but the mechanism is unclear. Recent studies have also reported the role and localization of PTN in mouse SSCs [51].SSC transplantation experiments showed that PTN increased the number of colonies, which is consistent with our in vitro experiments on human SSCs, which showed that PTN promoted the self-renewal and proliferation of SSCs. According to the authors’ immunofluorescence findings, PTN in the mouse testis primarily originated from Leydig cells and endothelial cells, and it coexisted with SDC4 in SSCs. However, our data suggested that PTN regulates human SSC development by binding to SDC2, which may result from species differences.

After SDC2 knockdown, we identified some differentially expressed genes using bulk RNA sequencing and analyzed their distribution in human testicular cells combined with scRNA data. SDC2 knockdown resulted in the down-regulation of GFRA1 and NUP88. Considering that the importance of GFRA1 for SSC self-renewal and proliferation is well recognized [34, 35, 52], we did not validate the effect of GFRA1 deficiency on SSCs. We still do not know the details of how PTN signaling regulates GFRA1 through SDC2, which requires further studies. NUP88 is a member of the nuclear pore complex (NUP) family, which mediates the transport of macromolecules across the nuclear membrane [53]. The build-up of NUP88 has been linked to various types of cancer, such as breast cancer [54], colorectal cancer [55], and skin cancer [56]. In human testis, NUP88 was highly expressed in spermatogonia and spermatocytes and regulated by SDC2 in SSCs. Given its involvement in promoting the growth of cancer cells, we speculated that it could potentially enhance the proliferation and self-renewal of SSCs. However, additional confirmation is required.

In a few patients with NOA, we found aberrant expression of PTN and SDC2, which were significantly down-regulated, especially in Spg MA and Spc MA testes, suggesting they might be associated with NOA. However, we have not yet identified pathogenic mutations of PTN and SDC2 in NOA patients. Due to the significant roles of PTN and SDC2 in the development of various organs and tissues, particularly in neural development [57], bone marrow [58] development, and angiogenesis [59], the presence of harmful mutations in these genes could hinder the development of multiple organs and tissues, potentially resulting in embryonic lethality. Consequently, the identification of mutations in NOA patients is impeded. In addition, we will expand the sample size and use conditional knockout mice to verify the role of PTN and SDC2 in regulating spermatogenesis under physiological conditions in future studies.

## Conclusion

**Fig. 7:**
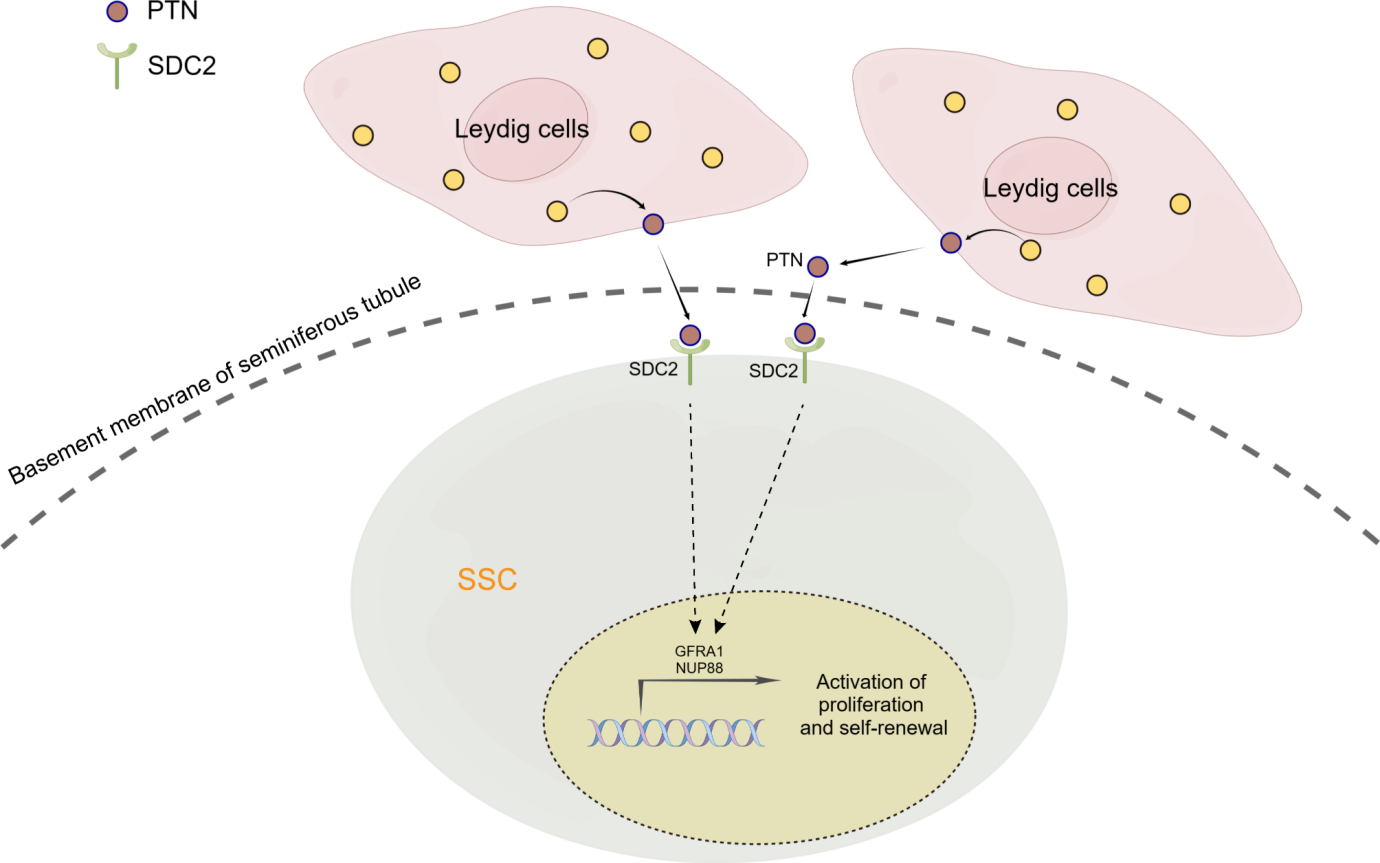
Graphic abstracts of this study. Using single-cell sequencing analysis and in vitro experiments, we found that Leydig cell-derived PTN promotes proliferation and self-renewal of human spermatogonial stem cells through binding to its ligand SDC2, and that their dysregulation may be associated with non-obstructive azoospermia

In summary, we show that PTN secreted by Leydig cells binds to SDC2 and regulates the growth and survival of SSCs via GFRA1 (Fig. 7). Altered expression of PTN and SDC2 may compromise spermatogenesis in NOA patients. Our findings shed light on the role of the testicular microenvironment in SSC fate specification and suggest a potential therapeutic avenue for male infertility.

## Electronic supplementary material

Below is the link to the electronic supplementary material.

Supplementary Material 1

Supplementary Material 2

## Data Availability

The information produced by this research can be acquired from the author in question upon a reasonable inquiry.

## Abbreviations

PTN: Pleiotrophin
SDC2: Syndecan-2
SSC: Spermatogonial Stem Cell
NOA: Non-Obstructive Azoospermia
OA: Obstructive Azoospermia
Diffing.Spg: Differentiating Spermatogonia
L: Leptotene Spermatocytes
Z: Zygotene Spermatocytes
P: Pachytene Spermatocytes
D: Diplotene Spermatocytes
RS: Round Spermatids
ES: Elongated Spermatids
LCs: Leydig Cells
SCs: Sertoli cells
ECs: Endothelial Cells
PTM: Peritubular Myoid Cells
Mø: Macrophages
Spg: Spermatogonia
Spc: Spermatocyte
HS: Hypospermatogenesis
MA: Maturation Arrest
