# Supplementary material for: PTN from Leydig cells activates SDC2 and modulates human spermatogonial stem cell proliferation and survival via GFRA1
Source: Biol Res. 2024 Sep 16;57:66. doi: 10.1186/s40659-024-00546-6 (PMC11406790; doi:10.1186/s40659-024-00546-6)
Supplement: Supplementary file 1 — Supplementary Material 1 [file 40659_2024_546_MOESM1_ESM.pdf]

Supplementary information

Niche-derived pleiotrophin regulates human spermatogonial stem cell proliferation and apoptosis through its receptor Syndecan-2

Supplemental Data:
3 Figures
3 Tables

Supplemental Figures

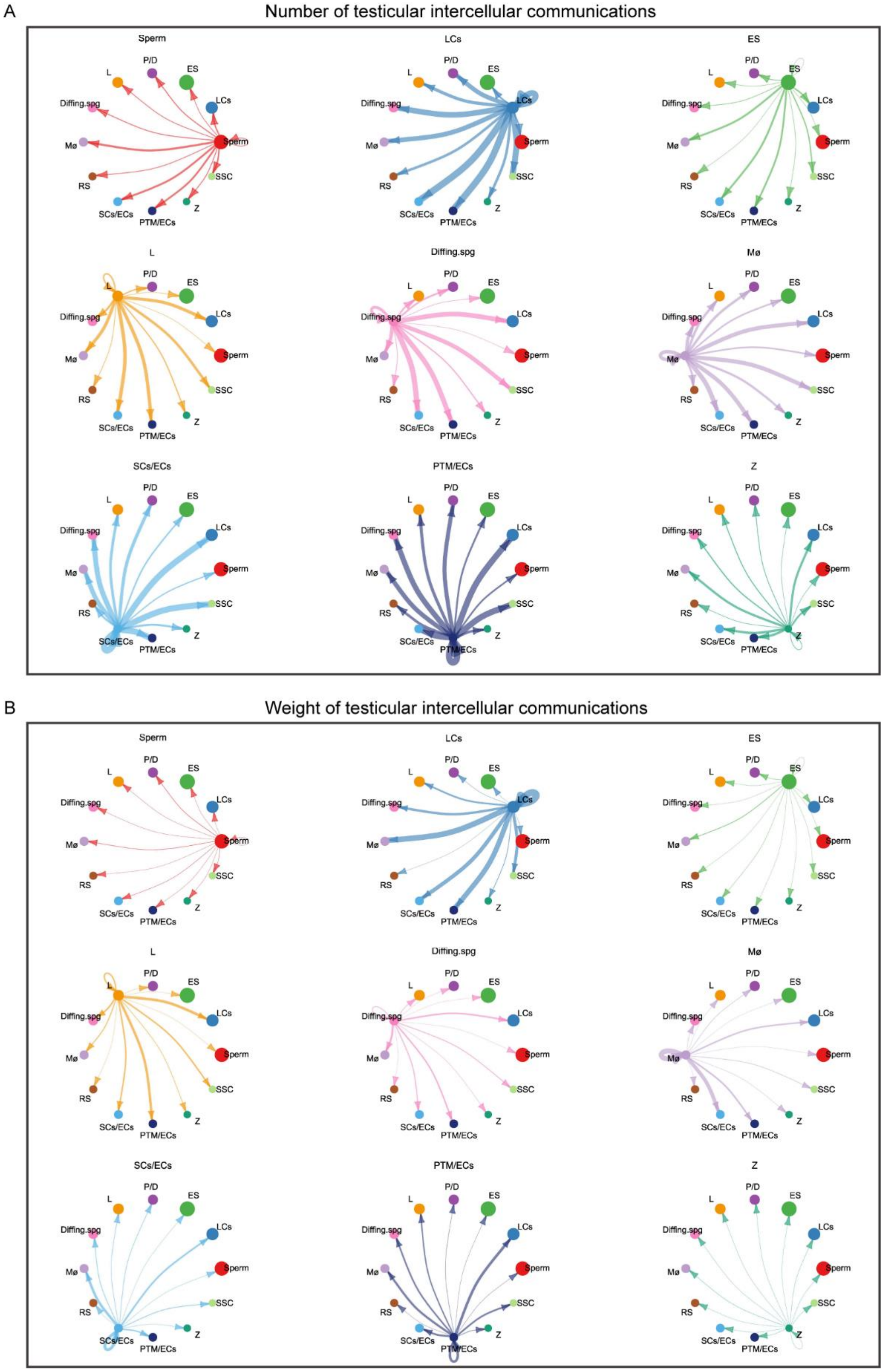

Figure S1. The number (A) and weight (B) of testicular cell interactions. The direction of the arrow represents the direction of cell signaling, The thickness of the arrows represents the number or weight of the signals.

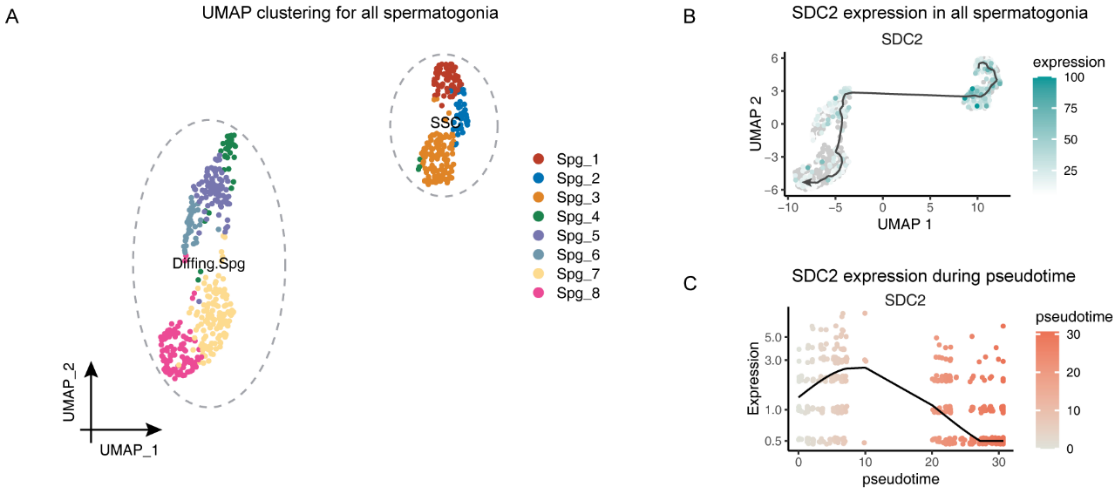

Figure S2. Expression of SDC2 along with spermatogonial differentiation trajectories (A) UMAP-based reclustering of spermatogonia, classifying all spermatogonia into eight subpopulations, including SSCs (Spg_1 to Spg_3) and differentiating spermatogonia (Spg_4 to Spg_8). (B) Heatmap of SDC2 expression along the spermatogonia developmental trajectory. (C) Mean levels of SDC2 during spermatogonia development.

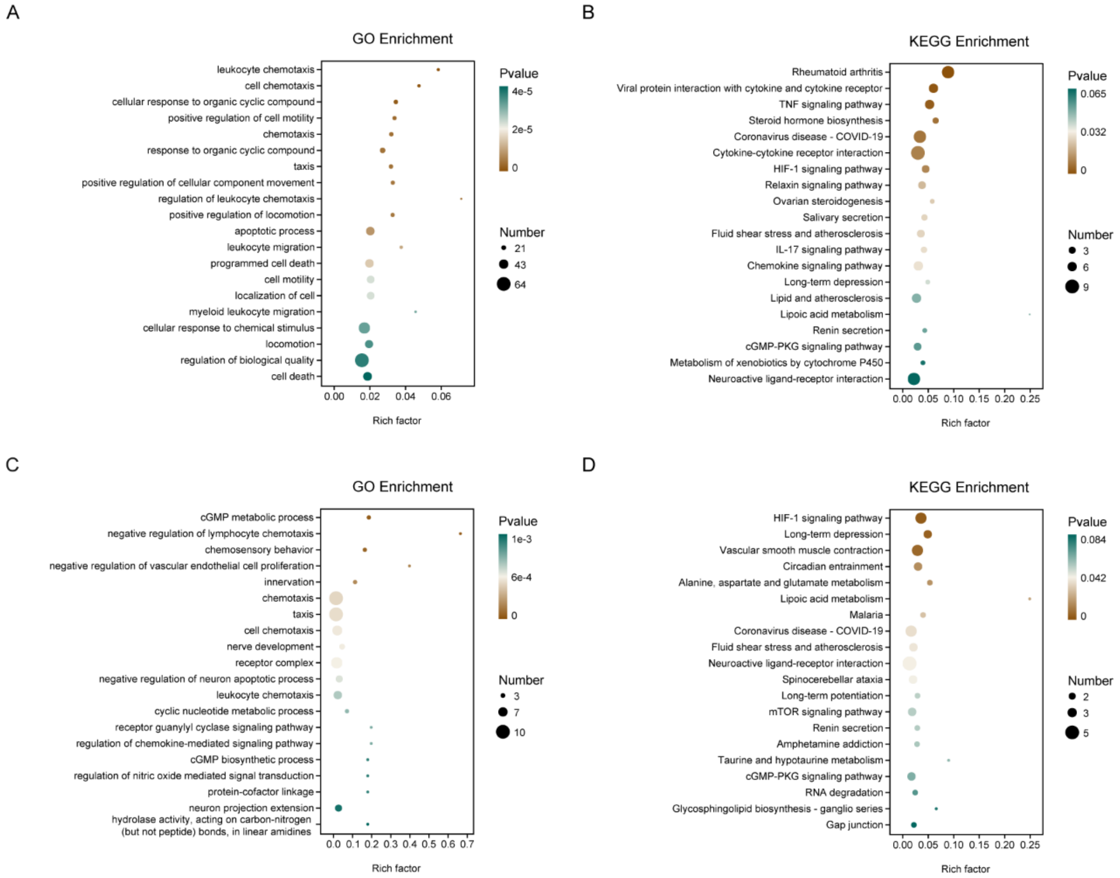

Figure S3. GO and KEGG enrichment. (A) GO enrichment for all DEGs. (B) KEGG enrichment for all DEGs. (C) GO enrichment for down-regulated genes. (D) KEGG enrichment for down-regulated genes.

Supplemental Tables

Table S1. The primers used for qPCR and RT-PCR

Genes	Sequence	Product sizes (bp)
SDC2	**F:** TGGAAACCACGACGCTGAATA<br>**R:** ATAACTCCACCAGCAATGACAG	208
GFRA1	**F:** CCAAAGGGAACAACTGCCTG<br>**R:** CGGTTGCAGACATCGTTGGA	121
NUP88	**F:** CCTGGCTTCCTAACCACGTC<br>**R:** AAGACCACGTTTCTCGTCAGC	139
EDN1	**F:** AAGGCAACAGACCGTGAAAAT<br>**R:** CGACCTGGTTTGTCTTAGGTG	237
SERPIND1	**F:** TTCCTCATCATAACATCTGCGTG<br>**R:** TGGAATCCAGTCGTTGGTGAC	183
EVI2B	**F:** ACCAACACAATTCAGCGACAC<br>**R:** GTTGTAGGCAAGTGGTTGTCC	142
KCNN4	**F:** CTGCTGCGTCTCTACCTGG<br>**R:** AGGGTGCGTGTTCATGTAAAG	144
SFN	**F:** ACTTTTCCGTCTTCCACTACGA<br>**R:** ACAGTGTCAGGTTGTCTCGC	165

Table S2. Antibodies applied in Western blots, immunofluorescence and immunoprecipitation

Antibodies	Source	Dilution	Incubation
Western blot			
SDC2	Sinobiological cat# 310259	1:1000	12h at 4℃
PLZF	SantaCruz cat#sc-28319	1:1000	12h at 4℃
PCNA	Abcam cat#ab29	1:500	12h at 4℃
CCNE1	Abcam cat#ab33911	1:1000	12h at 4℃
ACTB	Promab cat#20270	1:2000	12h at 4℃
GFRA1	R&D cat#AF560	1:500	12h at 4℃
PTN	SantaCruz cat#sc- 74443	1:500	12h at 4℃
Immunofluorescence			
SDC2	Sinobiological cat# 310259	1:50	16h at 4℃
GFRα1	R&D cat#AF560	1:25	16h at 4℃
PCNA	Abcam cat#ab29	1:50	16h at 4℃
KIT	R&D cat#AF332	1:25	16h at 4℃
PTN	SantaCruz cat#sc- 74443	1:50	16h at 4℃
Secondary Antibody			
Donkey anti-Rabbit IgG, Alexa Fluor 488	Thermo Fisher Scientific cat#A21206	1:1000	1h at 25℃
Donkey anti-Mouse IgG, Alexa Fluor	Thermo Fisher Scientific cat#A21203	1:1000	1h at 25℃

594			
Donkey anti-Goat IgG, Alexa Fluor 594	Thermo Fisher Scientific cat#A11058	1:1000	1h at 25°C

Table S3. The sequence of SDC2 small interfering RNA

**Names**	**Sense**	**Antisense**
SDC2-siRNA 1	GCUUCAGGAGUGUAUCCUAUUTT	AAUAGGAUACACUCCUGAAGCTT
SDC2-siRNA 2	GAAACCACGACGCUGAALATT	UAUUCAGCGUCGUGGUUUCTT
SDC2-siRNA 3	UGACCUUGGAGAACGCAAATT	UUUGCGUUCUCCAAGGUCATT
